# 

**Published:** 1885-09-20

**Authors:** 


☞ **Notice.**--- Subscribers in arrears are requested to remit their dues at once. Please take due notice and govern yourselves accordingly.

*RECEIPTED.*

1885—Drs. B. D. Caldwell, W. E. Quinn, I. F. Davis, F. H. Caldwell, to July; B. F. Darnell, to July; G. L. Landis, to Nov.

For 1884—Drs. M. Garrard, A. F. Durham, to July; M. C. Baldgridge, I, L. Cunningham, R. A. Pool, E. D. Chisolm, Marcus Powell, Wm. A. Joiner, M. E. Sproull, D. E. Kates, J. F. Brown, S. S. Johnson, Miles Johnson.

